# Targeting Ferroptosis in Glioblastoma: Molecular Mechanisms, Tumor Microenvironment, and Therapeutic Opportunities

**DOI:** 10.3390/cancers18122018

**Published:** 2026-06-22

**Authors:** Wiktoria Karło, Magdalena Długoń, Izabela Gutowska, Agata Wszołek, Wojciech Żwierełło

**Affiliations:** 1 Department of Medical Chemistry, Faculty of Pharmacy, Medical Biotechnology and Laboratory Medicine, Pomeranian Medical University in Szczecin, Powstańców Wielkopolskich 72, 70-111 Szczecin, Poland; 74482@student.pum.edu.pl (W.K.); 74037@student.pum.edu.pl (M.D.); wojciech.zwierello@pum.edu.pl (W.Ż.); 2 Institute of Biology, University of Szczecin, Felczaka 3c, 70-453 Szczecin, Poland; agata.wszolek@usz.edu.pl

**Keywords:** ferroptosis, glioma, glioblastoma, tumor microenvironment, oxidative stress, therapeutic resistance

## Abstract

Background: Glioblastoma (GBM) is the most aggressive primary brain tumor in adults and remains associated with poor prognosis despite multimodal treatment. Ferroptosis, an iron-dependent form of regulated cell death driven by lipid peroxidation and redox imbalance, has recently emerged as a potential therapeutic vulnerability in glioma. This review summarizes current knowledge on the molecular regulation of ferroptosis in glioma and discusses its implications for tumor progression, therapeutic resistance, and translational targeting. Methods: A structured narrative review of the literature was conducted using PubMed/MEDLINE, Scopus, and Web of Science databases. Experimental, translational, and clinically relevant studies investigating ferroptosis-related mechanisms and therapeutic strategies in glioma and GBM were qualitatively analyzed. Results: Ferroptosis in glioma is regulated by interconnected pathways involving iron metabolism, phospholipid remodeling, oxidative stress, and antioxidant defense systems, particularly the SLC7A11–glutathione–GPX4 axis. Additional protective mechanisms mediated by FSP1 and DHODH, together with regulatory networks involving NRF2, ATF4, p53, and hypoxia-related signaling, contribute to adaptive resistance to ferroptosis. Increasing evidence indicates that ferroptosis interacts bidirectionally with the glioma tumor microenvironment and may exert both antitumor and immunosuppressive effects. Preclinical studies further suggest that ferroptosis induction may enhance the efficacy of temozolomide, radiotherapy, and immunotherapy, although clinical translation remains limited by tumor heterogeneity, blood–brain barrier penetration, and resistance mechanisms. Conclusions: Ferroptosis represents a biologically plausible and therapeutically promising target in glioma. Improved understanding of ferroptosis regulation, tumor microenvironment interactions, and biomarker-guided therapeutic strategies may support the future development of more effective treatments for GBM.

## Simple Summary

Glioblastoma is the most aggressive primary brain tumor in adults and remains difficult to treat because of rapid growth, therapy resistance, and high recurrence rates. Ferroptosis is a recently recognized form of regulated cell death driven by iron accumulation, oxidative stress, and lipid damage within cellular membranes. Increasing evidence suggests that ferroptosis may influence glioblastoma progression and response to treatment. This review summarizes current knowledge on the molecular mechanisms regulating ferroptosis in glioma, including pathways involved in iron metabolism, antioxidant defense, and lipid peroxidation. Particular attention is given to the interaction between ferroptosis and the tumor microenvironment, as well as the potential use of ferroptosis-inducing compounds in combination with chemotherapy, radiotherapy, and immunotherapy. We also discuss major translational challenges, including drug resistance, tumor heterogeneity, and limited drug delivery to the brain. Understanding these mechanisms may support the development of novel therapeutic strategies for glioblastoma.

## 1. Introduction

Brain tumors are pathological cell masses that develop within the brain or its immediate surroundings. They constitute a diverse group of lesions that includes primary tumors arising from tissues of the central nervous system (CNS) and secondary tumors resulting from metastases originating in other organs. They may be benign or malignant. Their wide histological and clinical heterogeneity renders brain tumors particularly complex from a clinical perspective. The clinical significance of brain tumors results not only from their potentially aggressive course, but also from their location within an organ responsible for essential vital, cognitive, and neurological functions [1].

Glioma represent a heterogeneous group of primary CNS tumors and are among the most common malignant brain tumors in adults [1]. Adult diffuse gliomas encompass several tumor types, including glioblastoma (GBM), astrocytoma, and oligodendroglioma. These tumors differ in their histological appearance and molecular profile [2]. According to CBTRUS data from 2018 to 2022, the annual incidence of brain and other CNS tumors averaged 26.05 per 100,000 population. Nonmalignant lesions were diagnosed more frequently than malignant ones, and the overall incidence was higher among women. Glioma accounted for 22.2% of all CNS tumors. GBM was estimated to be the most frequently diagnosed malignant tumor, accounting for 13.7% of all tumors and 52.2% of malignant tumors [3]. Despite the progress made in recent years in the diagnosis and molecular classification of glioma, patient prognosis remains unsatisfactory in many cases, particularly in GBM. This tumor is characterized by marked biological aggressiveness, rapid growth, an infiltrative pattern, and a high risk of recurrence, all of which substantially limit long-term treatment efficacy [4]. This is reflected in the poor prognosis of patients with GBM, in whom median overall survival is approximately 15 months in many analyses, depending on factors such as patient age, performance status, extent of resection, and tumor molecular profile [5].

Despite the use of multimodal therapy including surgery, radiotherapy, and chemotherapy based mainly on temozolomide (TMZ), the effectiveness of glioma treatment remains limited. This is due, among other factors, to the infiltrative nature of tumor growth, the difficulty of achieving complete resection, substantial intratumoral heterogeneity, and a high tendency toward recurrence. Additional obstacles include the blood–brain barrier (BBB) and the immunosuppressive tumor microenvironment (TME), which reduce treatment efficacy and hinder the achievement of durable therapeutic responses. Given these limitations, increasing attention is being paid to biological mechanisms that may serve as novel therapeutic targets and enable the development of more effective treatment strategies for glioma [6].

Ferroptosis is a regulated, iron-dependent form of cell death whose key mechanism is the accumulation of toxic peroxidation products in phospholipids containing polyunsaturated fatty acids, leading to disruption of membrane integrity. Unlike other forms of cell death, its course is closely linked to disturbances in iron homeostasis, lipid metabolism, and redox balance [7]. This process is of particular interest in glioma research because glioma cells exhibit metabolic alterations that favor modulation of ferroptosis. Available studies indicate that attenuation of ferroptotic pathways may support the development and progression of glioma, whereas their activation is being considered a potential therapeutic strategy. Evidence increasingly suggests a role for ferroptosis in glioma resistance mechanisms to chemotherapy and radiotherapy [8]. In this context, discussion of the molecular mechanisms of ferroptosis and their role in glioma progression and treatment is of particular importance [9].

## 2. Search Strategy and Selection Criteria

The search strategy included combinations of the following terms: “ferroptosis”, “glioma”, “glioblastoma”, “GBM”, “SLC7A11”, “GPX4”, “FSP1”, “DHODH”, “iron metabolism”, “lipid peroxidation”, “tumor microenvironment”, “hypoxia”, “temozolomide”, and “radiotherapy”. The review primarily included articles published in English between 2009 and 2026, corresponding to the period following the characterization of ferroptosis as a distinct form of regulated cell death. Earlier studies were included when relevant to the biological background of iron metabolism, oxidative stress, or glioma biology.

Priority was given to original experimental studies performed in glioma or glioblastoma models, including in vitro, in vivo, and translational studies. Clinical studies, bioinformatic analyses, and high-quality review articles were also included when they provided mechanistic or therapeutic relevance. Studies from other tumor types were considered when glioma-specific evidence was limited but the described mechanisms were directly related to ferroptosis regulation or tumor microenvironment interactions.

The collected literature was analyzed qualitatively and organized into thematic sections addressing molecular mechanisms of ferroptosis, regulators of ferroptosis in glioma, interactions with the tumor microenvironment, ferroptosis-inducing compounds, combination therapies, and translational challenges. Because of the heterogeneity of experimental models and study designs, no quantitative meta-analysis was performed.

## 3. Mechanisms of Ferroptosis

Ferroptosis is an iron-dependent form of cell death in which enhanced lipid peroxidation is the central event. This phenomenon encompasses a series of interconnected biochemical reactions, ranging from accumulation of ferrous iron, reactive oxygen species (ROS) generation, and dysfunction of antioxidant systems to the peroxidation of membrane lipids [10].

### 3.1. Iron Accumulation

Iron homeostasis is tightly regulated to ensure an adequate supply of iron for biological processes while limiting toxicity resulting from its excess. Transferrin (Tf) plays the main role in iron transport by binding iron ions in the bloodstream and delivering them to cells. The transferrin–iron complex is recognized by transferrin receptor 1 (TfR1), enabling cellular uptake and increasing the intracellular iron pool [11]. After entering the cell, Fe^3+^ ions are reduced to Fe^2+^, the biologically active form of iron. Reduced Fe^2+^ ions are stored in ferritin or exported into the bloodstream via ferroportin. Under conditions of limited iron availability, ferritinophagy occurs in lysosomes in an ferritinophagy occurs in lysosomes in a nuclear receptor coactivator 4 (NCOA4)-dependent manner, leading to the release of stored iron. Disturbances in these mechanisms may result in expansion of the intracellular Fe^2+^ pool, thereby promoting iron accumulation and enhancing oxidative stress-related processes [12].

### 3.2. Lipid Peroxidation

Lipid peroxidation may be initiated in both nonenzymatic and enzymatic ways, and its extent depends largely on the availability of appropriate substrates within cellular membranes [13].

Free polyunsaturated fatty acids (PUFAs) do not exert direct pro-ferroptotic activity. In order to participate in peroxidation, they must first be activated and incorporated into phospholipids [10]. In the first step, acyl-CoA synthetase long-chain family member 4 (ACSL4) catalyzes the conjugation of long-chain PUFAs to coenzyme A, leading to the formation of acyl-CoA derivatives (PUFA-CoA). Subsequently, with the involvement of lysophosphatidylcholine acyltransferase 3 (LPCAT3) and other enzymes such as AGPAT3, these compounds are incorporated into membrane phospholipids, resulting in the generation of PUFA-PL species that are highly susceptible to reactive oxygen species [14].

One mechanism that initiates lipid peroxidation is the Fenton reaction, which occurs in the presence of Fe^2+^ ions [15]. In this process, hydrogen peroxide (H_2_O_2_) is converted into highly reactive hydroxyl radicals (•OH), which attack PUFA-PL and trigger chain lipid peroxidation [16]. This process includes an initiation phase, during which a phospholipid radical (PL•) is formed, followed by its reaction with oxygen to generate a phospholipid peroxyl radical (PLOO•). Propagation leads to the formation of phospholipid hydroperoxides (PLOOH) and additional radicals, enabling further continuation of the reaction. If protective mechanisms fail, PLOOH accumulates, cellular membranes are damaged, and the cell ultimately dies [13].

Lipid peroxidation may also be catalyzed by enzymes of the lipoxygenase (LOX) family. These include ALOX proteins, iron-containing enzymes that oxidize PUFAs and contribute to the generation of lipid hydroperoxides and increased oxidative stress [12,13]. ALOX enzymes have also been shown to directly oxidize PUFA-containing phospholipids (PUFA-PL), linking their activity to ferroptosis regulation. In addition, ALOX15 forms a complex with phosphatidylethanolamine-binding protein 1 (PEBP1), which intensifies lipid peroxidation and leads to the formation of 15-hydroperoxy-arachidonoyl-PE, a signal initiating ferroptosis [13].

### 3.3. The Cystine/Glutamate Antiporter System Xc^−^

A key role in controlling lipid peroxidation is also played by the cystine/glutamate antiporter system Xc^−^, which is responsible for transporting cystine into the cell. Solute carrier family 7 member 11 (SLC7A11), its major functional component, mediates cystine uptake, after which cystine is intracellularly reduced to cysteine, a substrate required for glutathione (GSH) synthesis [13,17]. GSH neutralizes reactive oxygen species and participates in the detoxification of harmful compounds [17]. In addition, it serves as a cofactor for glutathione peroxidase 4 (GPX4), which eliminates lipid peroxides by reducing them, with glutathione being oxidized in the process. In this way, GPX4 prevents accumulation of lipid peroxides and protects the cell from ferroptosis. High SLC7A11 expression supports redox homeostasis and strengthens cellular defense against oxidative stress. This phenomenon is observed in both tumor cells and tumor-associated immune cells, where it contributes to protection from ferroptosis induced by oxidative stress [16].

### 3.4. Alternative Protective Mechanisms

An alternative mechanism protecting cells from ferroptosis is based on the generation and regeneration of radical-trapping antioxidants (RTAs). Ferroptosis suppressor protein 1 (FSP1) plays a central role in this system by using NAD(P)H to reduce ubiquinone (coenzyme Q10, CoQ10) and vitamin K to their active antioxidant forms, CoQ10H2 and VKH2, respectively [18,19]. It has been shown that inhibition of ferroptosis by FSP1 is dependent on CoQ10, which acts as an antioxidant in phospholipids and lipoproteins [20]. The FSP1-dependent pathway complements GPX4 activity, forming an alternative protective system that shields the cell from ferroptosis [18]. This mechanism is particularly important when GPX4 activity is limited, because the anti-ferroptotic effect of FSP1 occurs independently of GPX4 expression [20].

## 4. Regulators of Ferroptosis in Glioma

**Figure 1:**
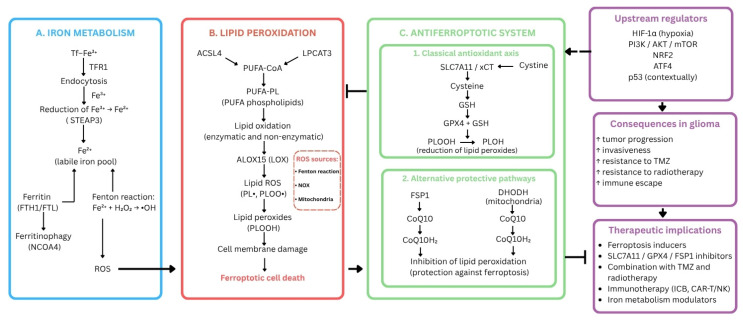
Molecular mechanisms regulating ferroptosis in glioma. Ferroptosis is regulated by the balance between iron-dependent ROS generation, lipid peroxidation, and antioxidant defense systems. Increased iron uptake through TFR1, NCOA4-mediated ferritinophagy, and Fenton chemistry expand the intracellular Fe^2+^ pool and promote oxidative stress. ACSL4- and LPCAT3-dependent incorporation of polyunsaturated fatty acids into membrane phospholipids enhances susceptibility to lipid peroxidation, ultimately leading to ferroptotic cell death. This process is counteracted primarily by the SLC7A11/xCT–GSH–GPX4 axis and by alternative protective pathways involving FSP1 and dihydroorotate dehydrogenase (DHODH). Upstream regulators such as NRF2, ATF4, p53, and hypoxia-related signaling modulate ferroptosis sensitivity in glioma. Dysregulation of these pathways contributes to tumor progression, treatment resistance, and immune modulation while also creating potential therapeutic opportunities.

The major molecular pathways regulating ferroptosis susceptibility, antioxidant defense, lipid peroxidation, and iron homeostasis in glioma are summarized in Figure 1.

### 4.1. Disturbances in Iron Homeostasis

Disturbances in iron metabolism are among the key factors modulating glioma cell susceptibility to ferroptosis. Expansion of the labile iron pool promotes oxidative stress and lipid peroxidation and may thereby increase the sensitivity of tumor cells to ferroptotic death [21]. Studies on brain tumors indicate that the expression of iron homeostasis-related genes such as TFRC, HAMP, HFE, and TFR2 may be altered in tumor tissue and astrocytoma cell lines, suggesting that dysregulation of iron metabolism contributes to the biology of these tumors [22].

Among iron-homeostasis regulators, TFRC is of particular importance in glioma because it mediates the uptake of transferrin-bound iron. In bioinformatic and validation analyses, TFRC was identified as one of the ferroptosis-related genes in glioma. Its expression was higher in more malignant glioma and correlated with poorer prognosis. It should be noted, however, that these findings are largely correlational and do not constitute direct evidence for a causal role of TFRC in glioma progression [23].

Ferritin is another important regulator of iron homeostasis in glioma. Ferritin heavy chain 1 (FTH1) may play a significant role in regulating glioma cell sensitivity to ferroptosis. In studies performed in U87 and U251 cells, dihydroartemisinin (DHA), while inducing ferroptosis, simultaneously increased FTH1 expression. This phenomenon was interpreted as an adaptive response of glioma cells that limits the extent of ferroptosis, supporting a protective role of FTH1 in reducing glioma cell sensitivity to ferroptotic death [24].

NCOA4, associated with ferritinophagy, is another important regulator of intracellular iron availability. Its activity increases the free iron pool and promotes ferroptosis. In studies of low-grade glioma, elevated NCOA4 expression was demonstrated, and functional analysis indicated its close association with autophagy and iron metabolism [25]. Similar observations were made in GBM cells, in which increased NCOA4 expression was associated with decreased FTH1 levels, increased Fe^2+^ concentration, and expansion of the labile iron pool, ultimately leading to enhanced lipid peroxidation and ferroptosis. Moreover, this process was shown to be modulated by TRIM7, which limits ferritinophagy and decreases susceptibility of GBM cells to ferroptosis through ubiquitination of NCOA4 [26].

### 4.2. Regulators of the SLC7A11–GSH–GPX4 Axis in Glioma

The SLC7A11–GSH–GPX4 axis is one of the key antioxidant systems limiting ferroptosis [27]. A crucial component of this axis is SLC7A11, the functional subunit of the system Xc^−^ antiporter [28]. In glioma, proper functioning of system Xc^−^ plays an essential role in maintaining redox homeostasis, and overexpression of SLC7A11 promotes GSH synthesis and reduces cellular susceptibility to ferroptosis. It has also been shown that high SLC7A11 expression may be associated with shorter patient survival, indicating its importance in glioma biology and aggressiveness [29]. SLC7A11 expression in glioma is regulated at multiple levels. Transcription factors such as ATF4 and NRF2 increase SLC7A11 expression, thereby supporting adaptation of tumor cells to oxidative stress, whereas p53 may act in the opposite direction by suppressing this pathway. Post-translational mechanisms have also been described, in which OTUB1 stabilizes SLC7A11 by limiting its degradation, thereby reducing glioma cell susceptibility to ferroptosis [30].

GSH is essential for maintaining redox homeostasis in glioma cells. Adequate GSH levels help limit oxidative damage and preserve GPX4 activity, which reduces phospholipid hydroperoxides and thereby suppresses ferroptosis [31]. GSH depletion or GPX4 inactivation leads to accumulation of PLOOH, which in the presence of Fe^2+^ undergoes further transformation, intensifying peroxidative processes and promoting ferroptosis [32]. Consequently, impaired GSH synthesis weakens antioxidant defense, increases the accumulation of lipid ROS, and enhances glioma cell susceptibility to ferroptosis [31]. GPX4 expression has been shown to be elevated in glioma tissues and cell lines, and its level increases with higher tumor grade. In some studies, high GPX4 expression was also associated with worse patient prognosis. Moreover, GPX4 silencing reduced glioma cell proliferation and migration and increased cell death, further confirming the importance of this enzyme in tumor progression [33].

Taken together, available findings indicate that the SLC7A11–GSH–GPX4 axis represents one of the main protective mechanisms limiting ferroptosis in glioma cells. Reduced expression of SLC7A11 and GPX4 is associated with increased ROS levels and enhanced ferroptosis, whereas their overexpression exerts a protective effect by attenuating this process. This supports the view that disruption of this axis may increase glioma cell sensitivity to ferroptotic death and may represent a potential therapeutic target [27].

### 4.3. Regulators of Lipid Metabolism and Lipid Peroxidation in Glioma

Lipid metabolism is an important determinant of glioma cell susceptibility to ferroptosis. In particular, regulators that affect membrane phospholipid composition and their susceptibility to peroxidation are of major significance. Enzymes such as ACSL4, LPCAT3, and LOX are involved in this process and promote ferroptosis [34].

ACSL4 is of particular importance in the regulation of lipid metabolism and lipid peroxidation in glioma. Studies have shown that ACSL4 expression is reduced in both glioma tissues and cell lines, which may indicate attenuation of a ferroptosis-prone phenotype. At the same time, ACSL4 overexpression enhanced ferroptosis, increased levels of lipid peroxidation products, and inhibited glioma cell proliferation, whereas silencing of this gene produced the opposite effect. Collectively, these findings support ACSL4 as an important regulator of ferroptosis in glioma and suggest that its activity may suppress tumor progression by enhancing susceptibility to lipid peroxidation [35].

In addition to factors that promote lipid peroxidation, membrane lipid-remodeling mechanisms that reduce susceptibility to ferroptosis may also play an important role. In this context, LPCAT1 is noteworthy because it increases the saturation degree of phospholipids by promoting incorporation of saturated fatty acids (SFAs) and reducing the proportion of PUFA-containing phospholipids. This leads to reduced lipid peroxidation and decreased susceptibility to ferroptosis. Experimental research indicates LPCAT1 overexpression reduced lipid peroxidation and increased resistance to ferroptosis inducers, whereas its silencing had the opposite effect by enhancing ferroptotic death. These findings indicate that remodeling of membrane phospholipid composition may represent an important adaptive mechanism limiting ferroptosis in tumor cells [36].

LPCAT3 plays a different role as a ferroptosis-related regulator. It participates in membrane phospholipid remodeling through incorporation of activated PUFAs into phospholipids. Analyses performed in low-grade glioma demonstrated that LPCAT3 belongs to a group of ferroptosis-related genes with potential prognostic significance. Its expression was elevated in both tumor tissue and glioma cell lines. Higher LPCAT3 levels were also associated with poor prognosis and with a positive correlation with CD274 expression, suggesting that this regulator may be involved not only in controlling ferroptosis susceptibility but also in shaping a more aggressive tumor phenotype [37].

In glioma, LOX enzymes may intensify lipid peroxidation and thereby increase cellular susceptibility to ferroptosis [38]. Particular attention has been paid to ALOX15, which in GBM was identified as one of the key genes driving ferroptosis. Higher ALOX15 expression was associated with activation of ferroptosis-related genes and increased lipid peroxidation. Importantly, reduced ALOX15 levels were observed more frequently in patients with recurrent glioma and were associated with shorter overall survival, supporting a potential tumor-suppressive role of ALOX15 mediated through ferroptosis-related lipid damage [39].

### 4.4. Transcription Factors and Signaling Pathways Modulating Ferroptosis in Glioma

Among the upstream regulators of ferroptosis in glioma, the transcription factor NRF2 plays a major role. NRF2 serves as a central regulator of cellular responses to oxidative stress. Under physiological conditions, it undergoes KEAP1-dependent degradation. Under oxidative stress, however, this process is inhibited, leading to NRF2 stabilization and activation of genes containing antioxidant response elements (AREs). As a result, NRF2 regulates the transcription of numerous genes involved in redox homeostasis, including SLC7A11 [40]. In studies performed in GBM cells, higher NRF2 expression co-occurred with increased levels of SLC7A11, HMOX1, and ABCC1, indicating its role in shaping a phenotype resistant to oxidative stress. Modulation of NRF2 activity was also shown to affect GSH levels and cellular sensitivity to ferroptosis, confirming its importance in ferroptosis regulation in glioma [41].

A similar protective effect has been attributed to ATF4. Studies have shown that ATF4 expression is increased in malignant glioma and is associated with a more aggressive tumor phenotype. ATF4 activation promotes xCT/SLC7A11 expression, whereas ATF4 silencing increases glioma cell sensitivity to ferroptosis inducers such as erastin, sorafenib, and RSL3 [42]. ATF4 and NRF2 are also thought to cooperate in regulating the expression of stress-response genes, and their coordinated effect on SLC7A11 enables tumor cells to adapt more effectively to oxidative and metabolic stress [40]. Collectively, these observations support a protective role of ATF4 against ferroptosis in glioma cells [42].

ATF3 may play an opposite role in the regulation of ferroptosis in glioma. Experiments found ATF3 can promote ferroptotic death of glioma cells by increasing accumulation of H_2_O_2_ and iron. This mechanism has been linked to induction of NOX4 and SOD1 together with decreased expression of xCT/SLC7A11 and catalase, resulting in reduced cysteine and GSH levels, intensified lipid peroxidation, and greater susceptibility of cells to ferroptosis. These findings suggest that ATF3 may function as a pro-ferroptotic regulator in glioma cells, acting differently from ATF4 and amplifying oxidative stress in tumor cells [43].

The complex nature of ferroptosis regulation in glioma is also illustrated by the role of p53. p53 has been shown to directly suppress SLC7A11 expression, thereby limiting cystine transport and reducing the ability of cells to maintain redox homeostasis. In addition, p53 may promote ferroptosis indirectly through mechanisms such as the SAT1/ALOX15 axis and activation of ALOX12, indicating that its effect encompasses both regulation of cystine metabolism and lipid peroxidation [44]. Accordingly, alterations in p53 activity may substantially modulate glioma cell sensitivity to ferroptosis in a molecular context-dependent manner. In glioma, the effects of p53 on ferroptosis may also be modulated by additional factors. RND1 has been reported to stabilize p53 and activate the p53–SLC7A11 axis, resulting in enhanced ferroptosis in GBM cells. In contrast, ALOXE3 deficiency may weaken the p53-dependent ferroptotic response, further emphasizing the complex and context-dependent nature of ferroptosis regulation in glioma [16]. These observations are complemented by data on p62, whose effects also depend on p53 status. In cells harboring mutant p53, p62 promotes ferroptosis and decreases SLC7A11 expression, whereas in cells with wild-type p53 it exerts the opposite effect. This suggests that p62 may modulate glioma cell susceptibility to ferroptosis in a context-dependent manner [45].

The PI3K/AKT/HIF-1α pathway also affects glioma sensitivity to ferroptosis. In studies performed in U87 and U251 cells, hypoxia weakened sulfasalazine-induced ferroptosis by increasing SLC7A11 expression. This effect was dependent on activation of the PI3K/AKT/HIF-1α pathway, because inhibition of AKT or HIF-1α reduced SLC7A11 levels and restored greater cellular sensitivity to ferroptosis. These findings suggest that the PI3K/AKT/HIF-1α pathway may contribute to glioma resistance to ferroptosis by enhancing the activity of the SLC7A11-dependent axis [46].

### 4.5. Alternative Regulators of Protection Against Ferroptosis in Glioma

One of the alternative regulators of protection against ferroptosis in glioma is FSP1, which acts in parallel with the SLC7A11–GSH–GPX4 axis and limits susceptibility to oxidative lipid damage. In glioma, special importance is attributed to mechanisms controlling FSP1 levels, because METTL3 has been shown to stabilize FSP1 mRNA in an m6A-dependent manner, thereby suppressing ferroptosis and supporting tumor progression. These observations suggest that FSP1 serves not only as an anti-ferroptotic effector but also as part of an adaptive survival program in glioma cells exposed to oxidative stress [47].

Dihydroorotate dehydrogenase, which acts in mitochondria, may also exert a protective role against ferroptosis. This enzyme helps limit lipid peroxidation within the mitochondrial membrane and thereby supports tumor-cell survival. Under conditions of reduced GPX4 activity, tumor cells become particularly dependent on DHODH as a mitochondrial ferroptosis checkpoint. Preclinical studies in GBM have further indicated that DHODH inhibition may increase cellular susceptibility to ferroptosis and potentially weaken treatment-resistance mechanisms, suggesting that this pathway may represent one of the adaptive mechanisms promoting tumor survival [48].

Importantly, both FSP1 and DHODH may function not only as protective effectors under basal conditions, but also as resistance mechanisms that allow glioma cells to survive GPX4-targeted therapy. When GPX4 activity is pharmacologically suppressed, tumor cells may shift their dependence toward FSP1- or DHODH-mediated lipid protection, thereby escaping ferroptotic death despite effective GPX4 inhibition. This suggests that simultaneous targeting of GPX4 and at least one of these alternative pathways may represent a promising strategy to achieve more sustained ferroptotic cell death in glioma. In line with this concept, selective inhibitors of FSP1 (such as iFSP1) and DHODH (such as brequinar) have been investigated in preclinical models as potential agents for overcoming ferroptosis resistance, although their application in glioma remains to be evaluated [49,50,51].

Key molecular regulators implicated in ferroptosis susceptibility, antioxidant defense, lipid metabolism, and iron homeostasis in glioma are summarized in Table 1.

## 5. Selected Interactions Between Ferroptosis and the Tumor Microenvironment

The tumor microenvironment of glioma is a complex cellular and biochemical ecosystem in which reciprocal interactions between tumor cells and nonmalignant components strongly shape tumor biology and disease course. The glioma TME comprises not only immune-cell populations but also vascular components, extracellular matrix elements, and soluble signaling molecules that together regulate tumor growth, therapeutic response, and cellular susceptibility to regulated forms of cell death. Hypoxia is of particular importance because it arises, among other reasons, from abnormal tumor vascularization. Activation of HIF-1α signaling may promote metabolic adaptation of GBM cells, angiogenesis, immunosuppression, and resistance to cell-death induction. In this context, ferroptosis remains dependent on microenvironmental conditions, because oxidative stress, iron metabolism, lipid peroxidation, and immune-cell activity can modulate its course and biological consequences [64].

**Figure 2:**
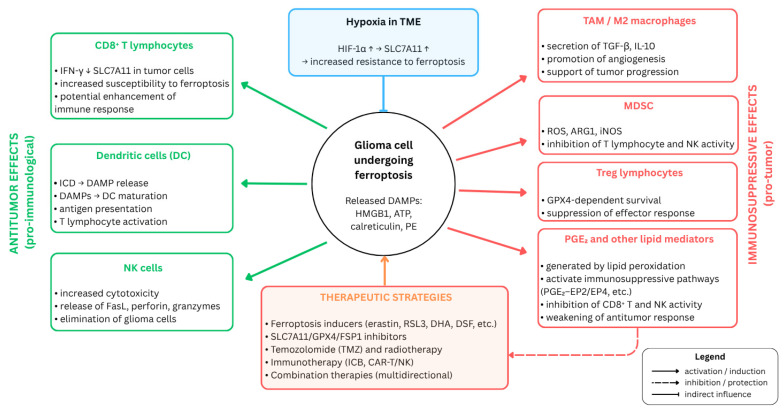
Interactions between ferroptosis and the glioma tumor microenvironment. Ferroptosis in glioma is closely linked to hypoxia, immune-cell activity, and immunosuppressive signaling within the tumor microenvironment (TME). Hypoxia-related HIF-1α signaling may increase SLC7A11 expression and enhance resistance to ferroptosis. Ferroptotic glioma cells release damage-associated molecular patterns (DAMPs), including HMGB1, ATP, calreticulin, and phosphatidylethanolamine (PE), which may stimulate dendritic cells, CD8^+^ T lymphocytes, and NK cells, thereby promoting antitumor immune responses. In parallel, immunosuppressive components of the TME, including TAMs/M2 macrophages, MDSCs, Treg lymphocytes, and lipid mediators such as PGE_2_, may suppress cytotoxic immune-cell activity and contribute to immune evasion. Potential therapeutic approaches include ferroptosis inducers, inhibition of antioxidant pathways, and combination strategies involving temozolomide, radiotherapy, and immunotherapy.

Among the most important cellular components of the TME are astrocytes, pericytes, endothelial cells, progenitor cells, and immune cells, including microglia and tumor-associated macrophages (TAMs), T lymphocytes such as helper (CD4^+^), cytotoxic (CD8^+^), and regulatory (Treg) T cells, myeloid-derived suppressor cells (MDSCs), NK cells, and dendritic cells (DCs). Together with extracellular matrix components, these cells form a dynamic environment that plays a central role in tumor growth, progression, and treatment response [65]. Glioma cells can actively recruit immune cells into the tumor niche by secreting cytokines such as TGF-β and GM-CSF and can subsequently promote their reprogramming into tumor-supportive phenotypes. Infiltrating immune cells may themselves secrete cytokines such as IL-1β and TGF-β, thereby intensifying the immunosuppressive character of the tumor microenvironment. This process, together with the presence of the BBB, hypoxia, and the acidic pH of the TME, may facilitate immune evasion by glioma cells [66]. In the immunosuppressive glioma microenvironment, ferroptosis may affect the relationship between tumor cells and immune components of the TME, including TAMs and T lymphocytes. However, the significance of this process remains complex and context-dependent, because iron metabolism, oxidative stress, and lipid peroxidation can bidirectionally modulate the antitumor immune response [67]. The major interactions between ferroptosis, hypoxia, immune-cell populations, and immunosuppressive mechanisms in the glioma tumor microenvironment are summarized in Figure 2.

### 5.1. Immune Modulation of Ferroptosis by TAMs and Microglia

TAMs are among the major immune components of the glioma microenvironment. They include both resident microglia-derived cells and macrophages originating from peripheral monocytes. These cells display substantial functional plasticity and may adopt diverse activation states, ranging from pro-inflammatory (M1-like) to immunosuppressive (M2-like). In GBM, many TAMs exhibit an M2-like phenotype that supports tumor progression, in part through secretion of immunosuppressive cytokines such as IL-10 and TGF-β [68].

Functional heterogeneity of TAMs and microglia may also be relevant in the context of ferroptosis, because their activation state influences their response to oxidative stress and lipid peroxidation. In a study performed in macrophage and microglial models, M2-like cells were found to be more susceptible to GPX4 inhibition-induced ferroptosis than M1-like cells. This relationship was attributed to higher iNOS expression and increased NO• production in M1-like cells, which limited the accumulation of pro-ferroptotic lipid peroxidation products and enhanced resistance to ferroptosis. Although this study did not directly concern glioma, its findings may suggest that the activation state of myeloid cells in the glioma TME influences their susceptibility to ferroptosis [69]. Ferroptosis may also affect communication between tumor cells and immune components of the TME. Tumor cells undergoing ferroptosis may release damage-associated molecular patterns (DAMPs) and other immunomodulatory signals, including HMGB1, calreticulin, ATP, and phosphatidylethanolamine, which may act on dendritic cells, macrophages, and CD8^+^ T lymphocytes. In glioma, however, the significance of this mechanism remains context-dependent, particularly because of the strongly immunosuppressive character of the TME [70]. At the same time, ferroptotic cells may release lipid mediators, including PGE2, which is regarded as an important immunosuppressive factor. PGE2 may weaken the function of NK cells and cytotoxic T lymphocytes, limit recruitment of cDC1 cells into the tumor niche, and suppress CD8^+^ T cell-dependent responses, thereby contributing to maintenance of an immunosuppressive tumor microenvironment [66].

Furthermore, an in vitro study using the human microglial cell line HMC3 showed that microglial ferroptosis may be intensified by disturbances in iron homeostasis and NCOA4-dependent ferritinophagy, leading to Fe^2+^ accumulation, increased lipid peroxidation, and reduced GPX4 and SLC7A11 expression [60].

### 5.2. Effects of Ferroptosis on Immune Cells in the Tumor Microenvironment

Regulatory T cells, MDSCs, NK cells, and dendritic cells are important immunologic components of the glioma TME and participate in shaping the antitumor response. Under conditions of profound immunosuppression, however, their function may be limited or reprogrammed in a manner that favors tumor progression. Ferroptosis may further affect this balance by modulating inflammatory signaling, immune-cell recruitment, and the activity of selected effector-cell populations [71,72].

MDSCs are among the key immunosuppressive populations in the glioma TME because they limit T-cell activity, weaken NK- and dendritic-cell function, and promote Treg expansion. These mechanisms involve, among others, production of reactive oxygen and nitrogen species, ARG1 and iNOS activity, disruption of L-arginine metabolism, and the action of immunosuppressive cytokines such as IL-10 and TGF-β. As a result, MDSCs contribute to weakening the cytotoxic immune response and maintaining an environment that favors glioma progression [73]. In the context of ferroptosis, the significance of MDSCs remains complex. A review on ferroptosis regulation in tumor and immune cells indicated that the neutrophil-like MDSC subtype may inhibit T-cell and NK-cell activity, partly through ROS production, and may also support Treg expansion. At the same time, these cells may possess mechanisms protecting them from ferroptosis, including ACOD1 activity and NRF2-dependent antioxidant responses, which may support maintenance of their immunosuppressive function. On the other hand, ferroptosis of these cells does not necessarily lead to a clearly beneficial antitumor effect, because it may be associated with release of PGE2 and oxidized PUFA phospholipids, both of which suppress innate and adaptive antitumor immunity. The authors also noted that MDSCs with high system Xc^−^ expression may contribute to T-cell inactivation by depleting the tumor microenvironment of cystine [74].

NK cells constitute a relatively small immune-cell population in the CNS and in glioma, but they participate in immune surveillance, regulation of inflammation, and direct elimination of tumor cells. Their activity in the GBM microenvironment is limited by numerous immunosuppressive mechanisms. Hypoxia may reduce NK-cell cytotoxicity through SHP-1-dependent attenuation of ERK and STAT3 phosphorylation, whereas TGF-β inhibits NK-cell activation and effector function by reducing the expression of activating receptors and suppressing mTOR signaling. In addition, glioma stem cells may reduce the expression of NKG2D ligands, weakening NK-dependent elimination of tumor cells. NK-cell function is also affected by GBM-derived exosomes, which may inhibit tumor-cell lysis, and by increased activity of the PD-1/PD-L1 axis, which promotes immune escape. With regard to ferroptosis, it has been suggested that its enhancement in glioma may potentially impair the antitumor cytotoxicity of immune cells. However, direct data concerning induction of ferroptosis in NK cells come from models of other cancers; therefore, in the case of GBM, the relationship between ferroptosis and NK-cell function should be regarded as a potential modulatory mechanism that requires further confirmation [75]. An example of the combination of ferroptosis induction with NK cell-dependent mechanisms is provided by the study of Hao et al., in which extracellular vesicles derived from NK cells were combined with RSL3-loaded liposomes. In C6 cells and in an orthotopic mouse glioma model, this approach enhanced ferroptosis of tumor cells, as demonstrated by reduced GPX4 and GSH levels together with increased ROS and lipid peroxidation. At the same time, NK cell-derived components supported the immune response through the action of FasL and IFN-γ, maturation of dendritic cells, and increased infiltration of CD4^+^ and CD8^+^ lymphocytes. These findings indicate that targeted induction of ferroptosis may cooperate with the immune response in the glioma microenvironment [76].

Dendritic cells are another important component of the antitumor response because they participate in antigen presentation and T-cell activation. In the glioma microenvironment, however, their function may be impaired by exosomes released by tumor cells. In the study by Yang et al., exosomes derived from GBM cells were taken up by mature dendritic cells and induced lipid accumulation, reduced viability, and decreased IL-12 secretion. At the same time, features of ferroptosis were observed, including increased Fe^2+^, ROS, MDA, and lipid peroxidation levels together with reduced NRF2, SLC7A11, and GPX4 expression. Importantly, inhibition of exosome release by glioma cells reduced lipid peroxidation in DCs, increased the number of DCs and CD8^+^ lymphocytes in the tumor microenvironment, and slowed glioma growth in an animal model. These findings indicate that ferroptosis of dendritic cells may constitute one mechanism of impaired antitumor immunity in the GBM microenvironment [77].

A possible contribution of Tregs to the immunosuppressive glioma microenvironment is suggested by analyses in low-grade glioma assessing the relationship between ferroptosis-related genes and both prognosis and TME characteristics. Wang et al. showed that patients in the high-risk group identified on the basis of a ferroptosis-related gene signature displayed a more immunosuppressive tumor microenvironment. This group was characterized by increased proportions of Tregs, M2 macrophages, and monocytes together with reduced CD8^+^ T-cell infiltration. The authors also reported increased expression of immune checkpoint genes and higher stromal and immune scores, suggesting a more complex and suppressive immune environment. These findings indicate that dysregulation of ferroptosis-related pathways in LGG may coexist with enhanced immunosuppression in the TME, including a greater Treg component [78]. In a review addressing ferroptosis in the immune microenvironment of glioma, system Xc^−^ was identified as an important link between ferroptosis regulation and immune-cell function. The authors cited data suggesting that disturbances in glutamate transport may enhance Treg function and contribute to GBM resistance to VEGF blockade. These observations underscore the importance of the GPX4-dependent pathway, including SLC7A11, GSH, and GPX4, in shaping the relationship between ferroptosis and the immunosuppressive glioma TME. GPX4 has also been proposed as a possible link between ferroptosis, chronic inflammation, and the immune response in glioma [66]. Additional mechanistic evidence regarding the role of GPX4 in Treg regulation is provided by studies by Xu et al. performed in models of other cancers. These authors demonstrated that GPX4 protects activated Tregs from excessive lipid peroxidation and ferroptosis, whereas loss of GPX4 weakens their immunosuppressive function. Treg-specific GPX4 deletion limited tumor growth and enhanced the antitumor response, including increased infiltration of tumor-infiltrating lymphocytes and increased IFN-γ and TNF-α production by CD8^+^ T cells. Because these studies were not conducted directly in glioma, the significance of Treg ferroptosis in the glioma TME still requires further confirmation [79]. Overall, although data from non-GBM cancer models provide valuable mechanistic insight into the relationship between ferroptosis and immune-cell function, further brain tumor-specific in vivo studies are essential to confirm whether these mechanisms operate identically within the GBM microenvironment.

### 5.3. Ferroptosis and CD8^+^ T Lymphocytes

CD8^+^ T lymphocytes are one of the main effector populations of the antitumor immune response because they can directly recognize and eliminate tumor cells. Traditionally, their activity has been associated primarily with perforin- and granzyme-dependent cytotoxic pathways and with Fas/FasL signaling. Wang et al., however, demonstrated that activated CD8^+^ T lymphocytes may also increase the susceptibility of tumor cells to ferroptosis. In these studies, immunotherapy, including PD-L1 blockade, enhanced lipid peroxidation in tumor cells, and the antitumor effect was weakened by ferroptosis inhibition. Mechanistically, a key role was attributed to IFN-γ secreted by CD8^+^ T cells, which reduced expression of SLC3A2 and SLC7A11, i.e., the subunits of the system Xc^−^ antiporter. This led to reduced cystine uptake, decreased GSH availability, and increased lipid peroxidation and ferroptosis in tumor cells. Furthermore, combining PD-L1 blockade with enzymatic depletion of cystine and cysteine using cyst(e)inase increased lipid peroxidation in tumor cells and enhanced T-cell responses in murine models. These data indicate that CD8^+^ T lymphocytes may support the antitumor response not only through classical cytotoxic mechanisms, but also by modulating tumor-cell susceptibility to ferroptosis. Although this study was not performed directly in a glioma model, the mechanism described may serve as a useful reference point for interpreting the relationship between CD8^+^ T cells and ferroptosis in the glioma TME [80].

Direct evidence regarding the effect of ferroptosis on T-cell infiltration in glioma was provided by the study of Schwantes et al. in a spheroid model of LN229 GBM cells. The authors showed that induction of ferroptosis with RSL3 led to increased lipid peroxidation and altered expression of ferroptosis-related genes such as SLC7A11, FTH, and TFR. After the addition of peripheral blood mononuclear cells (PBMCs), increased T-cell infiltration into ferroptotic spheroids was observed, involving both CD4^+^ and CD8^+^ cells, although the effect was stronger for CD4^+^ lymphocytes. Importantly, ferroptotic cells attracted T lymphocytes more effectively than apoptotic or necrotic cells, and this mechanism was linked to ATP release as a DAMP signal. ATP degradation by apyrase reduced T-cell infiltration, confirming the role of ATP-dependent signaling. At the same time, despite the greater extent of T-cell infiltration, contact with ferroptotic cells impaired T-cell activation, as evidenced by reduced secretion of IFN-γ, IL-2, and granzyme B after CD3/CD28 stimulation. These findings indicate that ferroptosis of GBM cells may increase recruitment of T lymphocytes, including CD8^+^ cells, but does not necessarily lead to full activation of the effector response [81].

### 5.4. Effect of Hypoxia on Glioma Cell Sensitivity to Ferroptosis

In addition to immune cells, physicochemical conditions within the glioma microenvironment, including hypoxia, are important factors that may alter the response of tumor cells to ferroptotic death. Hypoxia is one of the defining features of the glioma microenvironment and exerts a substantial effect on tumor-cell susceptibility to ferroptosis. In a study by Sun et al. conducted in U87 and U251 cells, hypoxia decreased sensitivity to sulfasalazine-induced ferroptosis, as reflected by increased IC50 values, reduced lipid peroxidation, and enhanced colony-forming capacity. This mechanism was linked to increased SLC7A11 expression under 1% O_2_ conditions, observed after as little as 6 h of hypoxic exposure. Silencing of SLC7A11, as well as inhibition of HIF-1α or AKT, reversed the protective effect of hypoxia and restored greater cellular susceptibility to ferroptosis. Moreover, in mouse models, the combination of the HIF-1α inhibitor PX-478 with sulfasalazine showed a stronger antitumor effect than sulfasalazine alone. These findings indicate that hypoxia in the glioma TME may promote ferroptosis resistance by strengthening the PI3K/AKT/HIF-1α/SLC7A11 axis [46].

The findings of Liu et al. suggest that hypoxia may also affect regulators of iron metabolism in glioma cells. The authors showed that expression of the ferritin light chain (FTL) was higher in high-grade glioma and was associated with poor prognosis. FTL positively correlated with HIF1A expression in glioma tissues, and in U87 and U251 cells its expression increased under hypoxic conditions in an HIF-1α-dependent manner. Mechanistically, HIF-1α was shown to bind directly to the HRE-3 sequence in the FTL promoter, thereby enhancing FTL expression. Although this study did not directly assess ferroptosis, it indicates that hypoxia may modulate iron metabolism in glioma cells and thereby indirectly influence a phenotype associated with tumor aggressiveness and treatment resistance [57].

Taken together, these findings suggest that hypoxia may reduce glioma cell sensitivity to ferroptosis by strengthening SLC7A11-dependent antioxidant mechanisms and by affecting elements of iron metabolism such as FTL.

## 6. Ferroptosis Inducers and Combination Strategies

Ferroptosis has attracted growing interest in oncologic research because of its antitumor properties, which has led to the identification of numerous compounds capable of inducing this form of cell death. Small molecules constitute the best-characterized group of ferroptosis inducers owing to their relative ease of characterization and potential for chemical modification. These compounds show considerable promise as candidates for novel anticancer therapies [82,83]. The SLC7A11–glutathione–GPX4 axis constitutes the primary molecular target of ferroptosis-based therapeutic strategies in glioma, and the mechanism of action of each compound discussed below can be understood in relation to its disruption of one or more components of this axis [31]. In glioma, ferroptosis inducers may be classified according to their mechanism of action into agents that inhibit GPX4 and agents that regulate the SLC7A11 system. In addition, compounds acting through lipid metabolism, ROS generation, and other regulatory pathways can also modulate redox homeostasis and glioma cell sensitivity to ferroptosis [29].

### 6.1. System Xc^−^ Inhibitors

One of the best-studied ferroptosis inducers is erastin, which triggers several molecular mechanisms. These include inhibition of cystine uptake through the system Xc^−^ antiporter, resulting in reduced GSH levels and enhanced oxidative stress. Consequently, GPX4 function becomes impaired, leading to accumulation of lipid peroxides and cell death through ferroptosis [82,84,85]. In addition, erastin inhibits voltage-dependent anion channels 2 (VDAC2), which leads to increased production of lipid ROS and non-apoptotic death of RAS-expressing tumor cells [82,86,87].

In vitro experiments found erastin induces ferroptosis in GBM cells through activation of the p53/p21 axis. The antitumor effect of this compound depends on the presence and functional activity of p53, as confirmed by the observation that inhibition of TP53 expression significantly reduces its efficacy. Moreover, erastin efficacy may be modulated by factors regulating the p53 pathway, including miR-491-5p [63]. In addition, studies have shown that erastin induces ferroptosis-associated changes in GBM cells, including mitochondrial remodeling and increased expression of proteins involved in iron metabolism. At the same time, its activity causes dose-dependent death of glioma cells, highlighting its potential as an antitumor agent [88]. Furthermore, in vitro studies in the U87 cell line demonstrated that erastin, through both ferroptosis and apoptosis induction, lowers galectin-9 transcription via extracellular acetyl-HMGB1, thereby inhibiting GBM cell invasion and proliferation [89].

Although erastin activity has been repeatedly confirmed in in vitro models, its therapeutic development remains limited because of poor in vivo efficacy. This has led to the development of new ferroptosis inducers, such as imidazole-based erastin derivatives, which are characterized by greater biological activity and improved metabolic stability, making them promising candidates for future use in GBM therapy [90].

Among clinically used drugs, sulfasalazine, commonly administered for rheumatoid arthritis, has also emerged as a potential ferroptosis inducer. Recent studies suggest that it may be considered as a potential anticancer agent in glioma therapy. Its mechanism of action is based on inhibition of the system Xc^−^ transporter, leading to reduced cystine uptake, decreased GSH synthesis, lower GPX4 activity, and enhanced lipid peroxidation [16,46]. Sulfasalazine also has a more favorable pharmacological profile than some other inducers, including fewer adverse effects and better solubility [46].

In vitro studies performed in the F98 and U251 cell lines showed that sulfasalazine exerts cytotoxic effects against glioma cells, while neurons display lower sensitivity to its activity. In contrast, in vivo studies indicated that sulfasalazine did not significantly affect tumor growth, but it did reduce peritumoral edema, resulting in smaller lesion volume. This suggests that sulfasalazine may function as a tumor microenvironment-normalizing agent despite limited cytotoxic activity [52]. It is also worth emphasizing that a substantial proportion of TMZ-induced DNA damage comprises N7-guanine and N3-adenine adducts, which are repaired primarily through the base excision repair (BER) pathway. Proficient BER activity may limit TMZ cytotoxicity by removing these lesions, whereas disruption of this pathway may potentiate drug efficacy by promoting the accumulation of DNA damage in tumor cells [91].

Because of poor brain penetration, sulfasalazine shows insufficient efficacy in malignant glioma [92]. For this reason, in vitro studies have demonstrated that combining sulfasalazine with DHA increases antitumor efficacy against glioma [93]. However, clinical studies confirming the possibility of repurposing sulfasalazine for GBM treatment are still lacking.

### 6.2. GPX4 Inhibitors

Another common ferroptosis inducer is RSL3, which binds to the selenocysteine residue of GPX4 and inhibits its activity [94]. Effective induction of ferroptosis by RSL3 is associated not only with GPX4 inhibition, but also with activation of the NF-κB pathway regulating ATF4 and SLC7A11 expression. The consequence of these processes is ROS accumulation and induction of ferroptosis in glioma cells [53].

Studies on RSL3 have shown that it induces ferroptosis-associated features in GBM cells, including mitochondrial ultrastructural changes and enhanced expression of markers related to this process. Moreover, its activity is dose-dependent [88]. Subsequent investigations demonstrated that RSL3 decreases the expression of ATF4 and SLC7A11 proteins without significantly affecting ATF4, SLC7A11, or HO-1 mRNA levels. This correlation points to post-translational regulation and enables effective induction of ferroptosis in glioblastoma cells. As a result, RSL3 inhibits proliferation of glioma cells in the U87 and U251 lines [53]. It has also been shown that RSL3 suppresses GBM growth both in vitro and in vivo. As a direct GPX4 inhibitor, it affects the regulation of genes associated with the cell cycle, ultimately limiting invasive growth of LN18 and LN229 glioma cells [54].

Despite these promising findings, RSL3 remains in the preclinical stage, mainly because of unfavorable pharmacokinetic properties and limited selectivity [90].

FIN56 is another ferroptosis inducer affecting GPX4. This compound promotes GPX4 degradation and binds to squalene synthase, resulting in depletion of endogenous CoQ10. Consequently, the FSP1/CoQ10 pathway becomes impaired, increasing cellular susceptibility to ferroptosis [56,70,82].

FIN56 inhibits GBM cell growth both in vitro and in vivo. In addition to inducing ferroptosis, this compound also triggers transcription factor EB-dependent lysosomal membrane permeabilization, leading to lysosome-dependent cell death. Moreover, its action results in reduced cell viability, inhibition of proliferation, and cell-cycle arrest in the LN229 and U118 cell lines [55].

The main limitation of FIN56 is its poor ability to cross the BBB. Therefore, research is being conducted on novel delivery systems that could enable effective use of ferroptosis inducers in glioma therapy. One example is a nanoparticle containing FIN56 (GDY-FIN56-RAP), which promotes GPX4 degradation and causes dose-dependent cytotoxicity in LN229 and T98G cell lines, confirming its effectiveness in inducing tumor-cell death [95]. Nevertheless, FIN56 still remains in the preclinical stage despite its high potential and attempts to improve its pharmacokinetic properties.

DHA is an active metabolite of artemisinin in vivo and exhibits several-fold greater biological activity than the parent compound [96]. Its mechanism of action is mainly related to reduced GPX4 expression while maintaining ACSL4 and xCT levels. This leads to increased total and lipid ROS, ultimately initiating glioma-cell death through ferroptosis. In addition, DHA has been shown to induce apoptosis and autophagy, cause cell-cycle arrest, and limit the invasive capacity of glioma cells [31,96]. DHA displays low cytotoxicity toward normal cells, selective antitumor activity, and a low tendency to induce drug resistance, making it a promising direction for cancer therapy research Despite its ability to induce ferroptosis in glioma cells, DHA simultaneously activates adaptive mechanisms that limit the effectiveness of this process. DHA-induced endoplasmic reticulum stress leads to activation of the PERK/ATF4/HSPA5 axis, which counteracts lipid peroxidation by increasing GPX4 expression and activity, thereby protecting cells from ferroptosis. This indicates that the pathway constitutes an important resistance mechanism to DHA-based therapy. Moreover, its inhibition using siRNA or small molecules increases the sensitivity of U251 and U373 glioma cells to DHA by enhancing ferroptosis, both in vitro and in vivo [62].

In U87 and U251 glioma cells treated with DHA, reduced GPX4 expression and increased HMOX1 expression were observed, both of which favor ferroptosis induction. Expression of FTH1, a negative regulator of this process, was also regulated. This mechanism is associated with the lncRNA TUG1/MAZ/FTH1 regulatory axis. Importantly, TUG1 overexpression or FTH1 inhibition enhanced the antitumor activity of DHA both in vitro and in vivo, suggesting a potential strategy for increasing the efficacy of ferroptosis-based therapy in glioma [24].

DHA has been investigated for many years and has been shown to cross the BBB in rats. Its relatively low water solubility favors membrane permeation and conjugation processes [58]. Nevertheless, DHA is still at the preclinical stage, although its properties suggest potential applicability in GBM therapy.

### 6.3. Ferroptosis Inducers Acting Through Other Mechanisms

Disulfiram (DSF), a thiuram derivative, exerts antitumor effects in glioma as an ROS-dependent ferroptosis inducer. Its mechanism of action includes enhancement of lipid peroxidation and ROS-dependent induction of lysosomal membrane permeabilization (LMP). Studies have shown that DSF increases ROS levels, reduces expression of key antioxidant proteins such as xCT and GPX4, and induces mitochondrial morphological changes characteristic of ferroptosis [16,97]. DSF has also been shown to reduce glioma-cell viability, inhibit proliferation, and induces cell-cycle arrest at the G0/G1 phase. These mechanisms result in suppressed growth of U251 and LN229 glioma cells in vitro [97].

DSF is characterized by favorable pharmacokinetic properties, including the ability to cross the BBB and a favorable safety profile. Preclinical studies and selected clinical studies suggest that DSF may show broad antitumor activity against various cancer types when administered with copper-containing supplements [98]. However, clinical findings remain inconclusive. In a randomized study in patients with recurrent GBM, DSF did not improve survival and was associated with increased toxicity [99]. By contrast, phase I/II clinical studies suggest potential efficacy in patients with BRAF-mutant GBM, supporting the need for further investigation [100] Table 2 and Table 3.

## 7. Combination Therapies

GBM remains a major therapeutic challenge because of its high degree of drug resistance. Despite advances in chemotherapy, targeted therapy, and immunotherapy, treatment outcomes remain unsatisfactory. The limited effectiveness of currently available methods is due to many factors, including impaired drug penetration across the BBB, substantial tumor heterogeneity, and TME-mediated immune evasion [103,104]. Ferroptosis represents a promising strategy for overcoming treatment resistance by targeting the Xc^−^/GSH/GPX4 axis [32]. Increasing evidence indicates that ferroptosis inducers may act synergistically with chemotherapy, immunotherapy, and radiotherapy, bypassing key resistance mechanisms [67].

### 7.1. Combined with Ferroptosis Induction

TMZ is the key chemotherapeutic agent used in glioma treatment. Resistance to TMZ is largely associated with the status of the DNA repair enzyme O6-methylguanine-DNA methyltransferase (MGMT). MGMT contributes to genomic stability by removing TMZ-induced DNA lesions. As a result, it neutralizes the effect of the drug and reduces its therapeutic efficacy, allowing continued replication of tumor cells despite the presence of DNA damage [104,105]. An important role in resistance is also played by disturbances in the mismatch repair (MMR) system, such as MSH6 deficiency or reduced MLH1 and PMS2 expression [104]. GBM cell lines exhibit considerable genetic heterogeneity, and therefore their molecular background may influence the response to therapeutic agents, including susceptibility to ferroptosis [106]. Likewise, in in vivo studies, the selection of an appropriate mouse model is of significant importance, as individual GBM models differ in biological characteristics that may affect the reliability and translatability of results concerning ferroptosis [107]. Molecular determinants such as EGFR, p53, MGMT, and IDH1 may be of particular relevance. Aberrant activation of EGFR signaling is associated with poorer prognosis in GBM and may modulate ferroptosis through effects on GSH metabolism [108]. The p53 status can regulate ferroptosis sensitivity in GBM cells through the p62-dependent p53/NRF2/SLC7A11 axis [45]. Furthermore, increased MGMT expression may limit the efficacy of TMZ, whereas IDH1 mutations, through the accumulation of 2-HG and associated alterations in cellular redox metabolism, may enhance the susceptibility of GBM cells to ferroptosis [109,110].

In contrast to classical therapies targeting DNA, alternative approaches such as ferroptosis induce cell death through iron-dependent lipid peroxidation, representing a distinct mechanism of action and a potential route for overcoming TMZ resistance [67].

It has been shown that glioma characterized by high xCT expression are more sensitive to combined erastin and TMZ treatment. This phenomenon is related to the action of erastin as an inhibitor of the xCT glutamate antiporter, which disrupts redox balance and increases cellular susceptibility to oxidative stress [111]. In addition, Zixiao Wang et al. demonstrated that the use of erastin in combination with TMZ in a hydrogel–liposomal nanoplatform enhances the therapeutic effect of TMZ. This formulation significantly improves the solubility of erastin, which is one of the major limitations of its clinical application. Therefore, a strategy combining both drugs and using advanced delivery systems may represent a promising therapeutic approach in glioma treatment [112]. At the same time, TMZ-resistant glioma cells with high NRF2 and GSH levels were shown to be sensitive to erastin. High expression of NRF2 and its target protein ABCC1 promotes increased susceptibility to ferroptosis, particularly under conditions of xCT inhibition by erastin. Importantly, despite elevated NRF2 and GSH levels, TMZ-resistant cells exhibit increased sensitivity to ferroptosis induction [41].

Synergistic activity has also been demonstrated for RSL3 and TMZ, resulting in inhibition of glioma-cell growth and invasiveness. This effect was observed both in vitro and in vivo, including in the presence of IDH1 mutation, suggesting broad therapeutic potential for this combination [54].

In in-vitro studies performed in the F98 and U251 cell lines, sulfasalazine was observed to be toxic to glioma cells and to enhance TMZ efficacy. This may result from activation of different cell-death pathways. Combination treatment proved more effective than monotherapy, suggesting that the two compounds act through separate mechanisms of cell death [52].

Synergistic activity with TMZ has also been demonstrated for haloperidol, an antipsychotic drug. Its action is based on indirect induction of ferroptosis, which increases TMZ efficacy both in vitro and in vivo. As a consequence, haloperidol sensitizes GBM cells to TMZ treatment [113].

Taken together, TMZ combined with ferroptosis inducers is more effective than monotherapy because it overcomes resistance mechanisms associated with the Xc^−^/GSH/GPX4 axis, disrupts redox homeostasis, and activates alternative cell-death pathways. This strategy shows therapeutic potential in GBM treatment, although additional studies are needed before it can be implemented clinically.

### 7.2. Radiotherapy Combined with Ferroptosis Induction

Radiotherapy is one of the core components of GBM treatment, but its effectiveness is limited by molecular alterations such as EGFR amplification, MGMT methylation, and TERT promoter mutations [61]. Hypoxia is also an important factor influencing radioresistance because it modifies ROS levels and increases the dependence of tumor cells on antioxidant systems [32,114,115].

Xuanzhong Wang et al. demonstrated that RSL3 increases glioma-cell sensitivity to ionizing radiation through multiple mechanisms. RSL3 inactivates GPX4, which enhances lipid peroxidation and increases radiosensitivity. At the same time, this compound inhibits transglutaminase 2-dependent DNA repair and epithelial–mesenchymal transition. As a consequence, RSL3 intensifies radiation-induced DNA double-strand breaks. These findings indicate that RSL3 may effectively overcome radioresistance and improve radiotherapy efficacy in glioma [116].

Studies on novel therapeutic strategies have also shown that the use of a FIN56-containing nanoparticle in combination with radiotherapy significantly increases treatment efficacy. This combination also prolonged survival in in vivo models, suggesting a potential role for FIN56 in overcoming radioresistance in glioma [96].

Sulfasalazine has been shown to act synergistically with irradiation under in vitro conditions, leading to enhanced DNA double-strand breaks and increased death of glioma cells. A similar effect was observed in vivo, where the combination of sulfasalazine with gamma knife radiosurgery prolonged survival in nude rats bearing human GBM xenografts [117].

In addition, DSF has been shown to increase tumor-cell sensitivity to irradiation, suggesting its potential use in combination with radiotherapy in GBM treatment [97].

### 7.3. Immunotherapy Combined with Ferroptosis Induction

One of the main challenges in GBM immunotherapy is therapeutic resistance and tumor heterogeneity. Combination strategies integrating immunotherapy with ferroptosis inducers may help counteract these limitations. The susceptibility of cells within the TME to ferroptosis is heterogeneous, and individual immune-cell populations may either promote or inhibit this process, translating into broad regulation of the immune response [118]. Ferroptosis induction may promote immunogenic cell death (ICD) through DAMP release, which may activate antitumor immune responses and thereby help counteract resistance to immunotherapy [119].

W. Wang et al. demonstrated that combining ferroptosis inducers with immune-checkpoint inhibitors enhances the immune response. CD8^+^ T lymphocytes activated by immunotherapy intensify lipid peroxidation in tumor cells, thereby increasing the effectiveness of the antitumor response. This mechanism is associated with IFN-γ secretion, which inhibits the xCT antiporter system and thereby induces ferroptosis [80]. These findings indicate the synergistic potential of combining immunotherapy with ferroptosis inducers. Moreover, in vitro studies have shown that erastin-induced ferroptosis leads to DAMP release from GBM tumor cells, which may be associated with improved responses to immunotherapy [120].

Research on combined immunotherapy and ferroptosis induction in the context of GBM remains limited and requires further investigation before this approach can be translated into clinical practice.

The main combination strategies involving ferroptosis induction in GBM are summarized in Table 4. Importantly, most available evidence remains preclinical or mechanistic, and the therapeutic relevance of these combinations in patients with GBM requires further validation in well-designed translational and clinical studies.

## 8. Challenges and Future Directions

### 8.1. Challenges

Ferroptosis inducers display antitumor activity in GBM, but their translation into clinical practice faces numerous limitations. The lack of a defined therapeutic threshold and the inability to monitor the course of ferroptosis hinder assessment of the risk of adverse effects, highlighting the need for further studies on safety [67,121,122]. In addition, ferroptosis inducers may exhibit dose-dependent neurotoxicity and may lead to DNA damage and injury to healthy cells [123,124]. One proposed approach to mitigating this off-target neurotoxicity is the concomitant use of ferroptosis inhibitors as neuroprotective agents, an option discussed further in Section 8.2. Effective application of ferroptosis-based therapy is also impeded by the BBB, which limits efficient delivery of therapeutic agents to the tumor site [125,126]. Therefore, strategies enabling effective BBB penetration, such as advanced drug-delivery systems or modification of compound pharmacokinetics, are urgently needed.

A further challenge lies in the unfavorable pharmacokinetic properties of ferroptosis inducers, including poor solubility and limited bioavailability, which reduce their systemic efficacy [10,124]. There is also difficulty in evaluating the effectiveness of ferroptosis under in vivo conditions. Current research relies largely on simplified cell-culture models that do not adequately reflect the biological and microenvironmental complexity of human GBM. Standardized animal models are needed if ferroptosis inducers are to be introduced into GBM anticancer therapy [10]. Another major obstacle is tumor heterogeneity, including genetic, metabolic, and epigenetic diversity, which results in variable susceptibility of tumor cells to ferroptosis induction. At the same time, there is a lack of reliable and validated biomarkers enabling precise patient selection. Consequently, there is a need to develop targeted therapeutic strategies based on appropriate biomarkers and patient stratification rather than uniform treatment schemes [121,127]. In addition, resistance to ferroptosis constitutes a major limitation to therapeutic application and may result from both TME-related features and cell-intrinsic mechanisms. TME remodeling that supports maintenance of redox balance, as well as heterogeneous responses to ferroptosis induction, make the therapeutic outcome difficult to predict [124,127]. Numerous resistance mechanisms also interfere with efficacy, including increased GPX4 expression, compensatory FSP1 activity, and the involvement of enzymes regulating lipid peroxidation [67,121]. Tumor cells may acquire resistance through activation of antioxidant systems, metabolic reprogramming, and genetic alterations [124].

### 8.2. Future Perspectives

Despite the many limitations associated with the use of ferroptosis inducers in GBM therapy, the rapid development of ferroptosis research points to several promising directions that may increase the therapeutic efficacy of this strategy. Despite these limitations, increasing evidence indicates that targeting adaptive antioxidant pathways, including FSP1/CoQ10 and DHODH, may improve the efficacy of ferroptosis-based therapies.

One of the main limitations of current studies is the predominant use of simplified in vitro and in vivo models that do not fully reflect the complexity of glioma. Future research should therefore implement more advanced and representative systems, such as GBM organoids and GBM-on-a-chip platforms, which more faithfully reproduce tumor heterogeneity. In addition, microfluidic technologies enable precise reconstruction of the TME, including regulation of fluid flow, nutrient gradients, and mechanical forces affecting cellular behavior. Combining organoids with microfluidic systems offers the possibility of developing more comprehensive research models that integrate the advantages of both approaches [128].

At present, research is increasingly focused on targeted delivery of ferroptosis inducers by means of nanotechnology [121]. This approach enables not only precise delivery of drugs to the tumor site, but also improvement of pharmacokinetic properties, including BBB penetration [16,67]. Through appropriate surface modifications, nanoparticles can selectively interact with tumor cells, potentially increasing therapeutic efficacy while reducing toxicity toward healthy tissues. In addition, they enable controlled and sustained release of therapeutic agents, which may help maintain effective concentrations within the tumor. Therefore, nanotechnology represents a promising strategy for enhancing the efficacy of ferroptosis-based therapy, particularly in GBM [16].

Another important research direction is the identification and validation of novel biomarkers for potential prognostic and predictive use in glioma. To this end, prognostic models based on the expression of ferroptosis-related genes are being developed and may support biomarker discovery [129]. Among the most frequently analyzed candidates are genes such as SLC7A11, GPX4, ACSL4, and FTH1 [54,130,131]. Increasing attention is also being paid to multigene ferroptosis signatures, which may better reflect the complex susceptibility of tumor cells to oxidative stress and lipid peroxidation [132]. In addition, lipid peroxidation markers including malondialdehyde (MDA) and 4-hydroxynonenal (4-HNE) are being evaluated, although their clinical utility remains limited because of insufficient specificity [59,133]. Despite the growing body of preclinical data, clinical use of ferroptosis biomarkers requires further validation, particularly in well-designed clinical trials. Their development may enable better patient stratification and support the implementation of ferroptosis-targeted therapeutic strategies.

It is also necessary to continue developing combination treatment strategies using ferroptosis inducers. GBM treatment is strongly limited by resistance to available therapies, whereas ferroptosis inducers show considerable potential in overcoming this resistance and improving treatment efficacy, especially when combined with nanotechnology-based solutions [121]. A particularly promising direction is the design of combination regimens showing synergistic activity between ferroptosis inducers and other treatment modalities. Expansion of current research toward clinical studies is also of crucial importance and may contribute to improved patient outcomes. In the longer term, the development of innovative, multidimensional therapeutic strategies will be necessary to address the complex challenges associated with GBM treatment.

Although ferroptosis induction remains the predominant ferroptosis-based therapeutic strategy explored in GBM, ferroptosis inhibitors are also important as mechanistic tools and potential cytoprotective agents. These compounds may inhibit ferroptosis by targeting different stages of the ferroptotic cascade, including iron metabolism, lipid peroxidation, and antioxidant defense. Iron chelators such as deferoxamine reduce the availability of re-dox-active iron and thereby limit Fenton reaction-driven ROS generation, whereas radical-trapping antioxidants, including ferrostatin-1 and liproxstatin-1, suppress lipid peroxidation and prevent the propagation of radical chain reactions within polyunsaturated fatty acid-containing membrane phospholipids [134]. Mechanistic studies further indicate that ferrostatin-1 and liproxstatin-1 act mainly as radical-trapping antioxidants that inhibit lipid peroxide accumulation and thereby prevent ferroptotic cell death [135]. However, their therapeutic application in GBM remains limited and context-dependent, because excessive inhibition of ferroptosis could counteract strategies aimed at eliminating therapy-resistant tumor cells. Nevertheless, ferroptosis inhibitors may be valuable as cyto-protective modulators, particularly for reducing oxidative damage and ferroptosis-related injury in nonmalignant neural cells during aggressive combination therapies [136]. The therapeutic application of ferroptosis inhibitors in GBM remains limited and context-dependent. Since ferroptosis induction is increasingly considered a strategy to overcome therapeutic resistance in GBM, excessive or nonselective inhibition of ferroptosis could potentially counteract approaches aimed at eliminating therapy-resistant tumor cells [122].

Given the potential of ferroptosis in GBM therapy, future studies should also focus on reducing the toxicity of the compounds used and on developing effective methods for therapy monitoring [126]. Improving the safety, selectivity, and monitoring of ferroptosis-targeted therapies will be essential for their successful clinical translation in GBM.

## 9. Conclusions

This review has several limitations that should be acknowledged. First, a substantial proportion of the available data on ferroptosis in glioma comes from in vitro studies and animal models, which do not fully reflect the cellular heterogeneity and microenvironmental complexity of human GBM. Second, many of the relationships presented are based on correlational analyses, and their causal importance requires further experimental validation. Third, variability in experimental conditions, including differences in the cell lines used, ferroptosis inducers, and analytical methods, limits comparability across studies. In addition, the translational potential of ferroptosis-targeted strategies remains constrained by difficulties associated with drug delivery across the BBB, possible systemic toxicity, and the development of adaptive resistance mechanisms. Finally, the absence of standardized biomarkers hinders patient stratification and the implementation of ferroptosis-based therapies in clinical practice.

Ferroptosis is a regulated mechanism of cell death whose significance in the biology of brain tumors, especially glioma, is being investigated with increasing intensity. Its induction and progression are associated with disturbances in iron homeostasis, enhanced lipid peroxidation, and failure of antioxidant systems, including the SLC7A11–GSH–GPX4 axis. In light of the currently available data, this process may play a context-dependent, bidirectional role in glioma. On the one hand, reduced susceptibility of tumor cells to ferroptosis may promote their survival, tumor progression, and the development of therapeutic resistance. On the other hand, pharmacological induction of ferroptosis is considered a potential strategy for enhancing the efficacy of anticancer treatment. The susceptibility of glioma cells to ferroptosis may additionally depend on the molecular profile of the tumor, including IDH1 mutation status, EGFR amplification, and MGMT expression.

Available studies indicate that numerous molecular factors are involved in the regulation of ferroptosis in glioma, including regulators of iron metabolism, proteins responsible for membrane phospholipid remodeling, transcription factors, and alternative systems protecting against lipid peroxidation. Among the most frequently described regulators of ferroptosis in glioma are TFRC, NCOA4, FTH1, ACSL4, LPCAT3, ALOX15, NRF2, ATF4, p53, FSP1, and DHODH. Their activity may influence the balance between tumor-cell survival and induction of ferroptotic death. It should be emphasized, however, that some of the available observations are correlational or derived from preclinical models, and therefore their biological and clinical relevance requires further validation, particularly in clinical studies.

The interaction between ferroptosis and the tumor microenvironment is bidirectional and context-dependent. Modulation of the function of tumor-associated macrophages, microglia, MDSCs, CD8^+^ T lymphocytes, NK cells, dendritic cells, and regulatory T cells, as well as the effect of hypoxia on the PI3K/AKT/HIF-1α/SLC7A11 axis, indicate that ferroptosis may either support the antitumor response or contribute to maintenance of an immunosuppressive TME phenotype. These findings highlight the need for further investigation of ferroptosis within the complex glioma microenvironment. The effect of ferroptosis in the tumor microenvironment may be ambivalent, because in addition to activating immune responses, it may also promote immunosuppression through the release of lipid mediators.

Among the compounds inducing ferroptosis in GBM, particular importance is attributed to system Xc^−^ inhibitors such as erastin and sulfasalazine, GPX4 inhibitors such as RSL3 and FIN56, as well as dihydroartemisinin and disulfiram. Their antitumor effects have been demonstrated in numerous preclinical studies, both in vitro and in vivo. Despite these encouraging results, application of these compounds in clinical practice remains limited. This is due, among other reasons, to unfavorable pharmacokinetic properties, limited ability to cross the BBB, potential neurotoxicity, and resistance mechanisms in tumor cells. Combination therapies are of particular interest, especially those in which ferroptosis inducers are combined with temozolomide, radiotherapy, or immunotherapy. Such an approach may increase treatment efficacy and help overcome glioma resistance.

In summary, ferroptosis represents a biologically well-founded and potentially promising therapeutic target in glioma treatment, and its integration with classical treatment regimens may become an important component of strategies aimed at improving the prognosis of patients with GBM. Continued translational research and the design of clinical trials remain essential in order to verify the efficacy and safety of this approach in oncologic practice and to develop individualized therapeutic regimens based on the molecular profile of the tumor and the characteristics of the tumor microenvironment.

## Figures and Tables

**Table 1:** Key Regulators of Ferroptosis in Glioma.

Regulator/ Pathway	Function in Ferroptosis	Relevance in Glioma	Refs.
SLC7A11 (xCT)	Cystine transport; GSH synthesis	Protection against ferroptosis; therapeutic resistance	[27,28,29,30,40,46,52]
GPX4	Reduction in PLOOH	Survival, proliferation, and migration of glioma cells	[31,32,33,53,54,55]
FSP1/CoQ10	GPX4-independent lipid protection	Adaptation to oxidative stress	[18,19,20,47,56]
DHODH	Mitochondrial CoQ reduction	Mitochondrial ferroptosis checkpoint	[20,48]
TFRC	Iron uptake	Higher expression in more malignant glioma	[22,23]
FTH1/FTL	Iron storage	Limitation of the Fe^2+^ pool; resistance to ferroptosis	[24,54,57,58,59]
NCOA4	Ferritinophagy	Increased Fe^2+^ and enhanced ferroptosis	[25,26,60]
ACSL4	PUFA activation	Greater susceptibility to lipid peroxidation	[34,35]
LPCAT1/LPCAT3	Phospholipid remodeling	Regulation of membrane susceptibility to ferroptosis	[36,37]
ALOX15/LOX	Enzymatic lipid peroxidation	Pro-ferroptotic effect; possible association with prognosis	[13,38,39]
NRF2	Antioxidant response	Redox adaptation; involvement in resistance	[40,41,61]
ATF4	Stress response and SLC7A11 regulation	Protection against ferroptosis	[40,42,53,62]
ATF3	Amplification of oxidative stress	Pro-ferroptotic regulation	[43]
p53	Regulation of SLC7A11 and lipid metabolism	Effect dependent on molecular context	[44,45,63]
PI3K/AKT/HIF-1α	Adaptation to hypoxia	Increased SLC7A11; resistance to ferroptosis	[46,57]

**Table 2:** Summary of key ferroptosis inducers investigated in glioblastoma: mechanism of action, BBB permeability, and research stage.

Drug	Mechanism of Action (Target)	BBB Permeability	Research Stage (GBM)
Erastin	Inhibition of system Xc^−^ (SLC7A11/SLC3A2); cystine deprivation; GSH depletion; secondary impairment of GPX4 activity	Poor (limited in vivo efficacy)	Preclinical (in vitro; in vivo limited)
Sulfasalazine	Inhibition of system Xc^−^ (SLC7A11); GSH depletion; GPX4 inactivation	Poor (insufficient brain penetration)	Preclinical (in vitro/in vivo); no GBM clinical trials confirmed
RSL3	Direct GPX4 inhibition (selenocysteine binding)	Not established (unfavorable pharmacokinetics)	Preclinical (in vitro/in vivo)
FIN56	GPX4 degradation; activation of squalene synthase; depletion of CoQ10	Poor (nanoparticle delivery under investigation)	Preclinical (in vitro/in vivo)
Dihydroartemisinin (DHA)	GPX4 downregulation; ROS/lipid ROS accumulation; apoptosis and autophagy induction	Crosses BBB (demonstrated in rats)	Preclinical (in vitro/in vivo)
Disulfiram (DSF)	ROS-dependent ferroptosis; LMP induction; xCT and GPX4 downregulation	Crosses BBB (favorable pharmacokinetics)	Clinical (Phase I/II in BRAF-mutant GBM; Phase III negative in recurrent GBM [99,100])

Legend: 1. BBB, blood–brain barrier; CoQ10, coenzyme Q10; DSF, disulfiram; DHA, dihydroartemisinin; GPX4, glutathione peroxidase 4; GSH, glutathione; LMP, lysosomal membrane permeabilization; ROS, reactive oxygen species; SLC7A11, solute carrier family 7 member 11; xCT, cystine/glutamate antiporter light chain.

**Table 3:** Clinical and late-stage preclinical studies of ferroptosis-related therapies in glioblastoma registered in clinical trial databases.

Drug/ Intervention	Mechanism (Ferroptosis-Related)	Trial ID/ Registry	Phase	Patient Population	Key Findings/ Status
Disulfiram + Copper (DSF/Cu)	ROS-dependent ferroptosis; xCT and GPX4 downregulation; LMP induction	NCT02678975 (DIRECT trial) EudraCT: 2016-000167-16	Phase II/III, (randomized, multicenter, open-label)	Recurrent GBM (1st recurrence; *n* = 88)	No survival benefit vs. SOC (mOS: 5.5 vs. 8.2 months); increased Grade ≥3 toxicity (34% vs. 11%); Completed/final results published [99]
Disulfiram + Copper + RT + Temozolomide	ROS-dependent ferroptosis; xCT and GPX4 downregulation	NCT02715609	Phase I/II	Newly diagnosed GBM (*n* = 33)	MTD: 375 mg/day DSF; recommended Phase II dose: 250 mg/day; limited efficacy overall; promising signals in BRAF-mutant GBM (*n* = 3); CuET not detected in tumor tissue; Completed [100]
Sulfasalazine + Gamma Knife Radiosurgery	System Xc^−^ inhibition; GSH depletion; GPX4 inactivation	NCT04205357 (SAS-GKRS)	Phase I	Recurrent GBM	Safety and feasibility study; Completed (2020–2022); Completed; phase I safety data published in 2026. [101]
Sulfasalazine (monotherapy/ combination)	System Xc^−^ inhibition; GSH depletion; NF-κB/IKK inhibition	ISRCTN45828668 (EudraCT 2004-004392-11)	Phase I/II (randomized)	Recurrent/ progressing malignant glioma	Terminated after interim analysis due to serious adverse events and lack of objective clinical response; no confirmed GBM-specific clinical benefit [102]

Legend 2. BBB, blood–brain barrier; CuET, diethyl-dithiocarbamate-copper complex; DSF, disulfiram; GBM, glioblastoma; GPX4, glutathione peroxidase 4; GSH, glutathione; LMP, lysosomal membrane permeabilization; mOS, median overall survival; MTD, maximum tolerated dose; ROS, reactive oxygen species; RT, radiotherapy; SOC, standard of care; TMZ, temozolomide; xCT/SLC7A11, cystine/glutamate antiporter. References in brackets correspond to citations in the main text.

**Table 4:** Combination strategies pairing ferroptosis induction with temozolomide, radiotherapy, or immunotherapy in glioblastoma: coordinated effect, key outcomes, limitations, and development stage.

Ferroptosis Agent (Target)	Combination Partner	Coordinated Effect/Rationale	Key Outcomes	Limitations	Development Stage (Refs.)
Erastin (system Xc^−^/SLC7A11 inhibition)	TMZ	Enhances TMZ sensitivity in xCT-high glioma; GSH depletion amplifies oxidative stress and overcomes Xc^−^/GSH/GPX4-dependent resistance	xCT-high glioma show greater sensitivity to combined erastin + TMZ; TMZ-resistant cells with high NRF2/GSH remain sensitive to erastin, suggesting that xCT inhibition can overcome NRF2-associated antioxidant defenses	In vitro evidence only; erastin shows poor in vivo efficacy and low solubility	Preclinical, in vitro [41,111]
Erastin (Xc^−^ inhibition; hydrogel–liposomal nanoplatform)	TMZ	Co-delivery improves erastin solubility and potentiates the TMZ effect	Nanoplatform enhanced the therapeutic effect relative to TMZ alone and improved erastin solubility	Early stage delivery platform; no clinical data	Preclinical, in vitro/in vivo [112]
RSL3 (direct GPX4 inhibition)	TMZ	Synergistic suppression of tumor growth and invasiveness through GPX4 inactivation	Synergy demonstrated in vitro and in vivo, including in the IDH1-mutant context	RSL3 has unfavorable pharmacokinetics and limited selectivity; preclinical	Preclinical, in vitro/in vivo [54]
Sulfasalazine (system Xc^−^ inhibition)	TMZ	Enhances TMZ efficacy by activating a distinct, ferroptosis-related cell-death pathway	Combination more effective than monotherapy in F98 and U251 cells, consistent with independent mechanisms of cell death	Poor brain penetration; no GBM clinical confirmation	Preclinical, in vitro [52]
Haloperidol (indirect ferroptosis induction)	TMZ	Sensitizes GBM cells to TMZ via indirect ferroptosis induction	Increased TMZ efficacy in vitro and in vivo	Indirect, incompletely defined mechanism; preclinical	Preclinical, in vitro/in vivo [113]
RSL3 (direct GPX4 inhibition)	Radiotherapy	Increases radiosensitivity through GPX4 inhibition and ferroptosis induction; additional suppression of transglutaminase 2-associated DNA repair and epithelial–mesenchymal transition has been reported	Intensified radiation-induced DNA double-strand breaks; counteracts radioresistance	Preclinical; RSL3 pharmacokinetic limitations	Preclinical [116]
FIN56 (GPX4 degradation; nanoparticle)	Radiotherapy	Nanoparticle-delivered GPX4 degradation enhances radiotherapy efficacy	Increased treatment efficacy and prolonged survival in vivo	Poor BBB penetration addressed only via nanoparticle delivery; preclinical	Preclinical, in vivo [96]
Sulfasalazine (system Xc^−^ inhibition)	Radiotherapy (gamma knife)	Xc^−^ inhibition synergizes with irradiation, enhancing DNA double-strand breaks	Increased glioma-cell death in vitro; prolonged survival in human GBM xenografts	Poor brain penetration; no clinical confirmation	Preclinical, in vitro/in vivo [117]
Disulfiram (ROS-dependent ferroptosis)	Radiotherapy	Increases tumor-cell radiosensitivity through ROS-mediated oxidative stress and ferroptosis-associated mechanisms	Enhanced sensitivity to irradiation	Preliminary evidence; clinical disulfiram data inconsistent	Preclinical [97]
Ferroptosis inducers (broad; Xc^−^/GPX4 axis)	Immunotherapy (immune-checkpoint inhibition; anti–PD-L1)	CD8^+^ T cell-derived IFN-γ suppresses xCT and drives tumor-cell ferroptosis; ferroptotic immunogenic cell death amplifies the response	Immunotherapy-activated CD8^+^ T cells intensified lipid peroxidation, producing a synergistic antitumor response	Mechanistic data largely from non-GBM models; strong GBM immunosuppression	Preliminary/preclinical evidence; clinical DSF data in GBM remain inconsistent [120].
Erastin (Xc^−^ inhibition)	Immunotherapy	Erastin-induced ferroptosis promotes DAMP release (immunogenic cell death)	DAMP release from GBM cells in vitro, potentially immunogenic in vitro effect	In vitro association only	Preclinical, in vitro [120]

Legend 3. BBB, blood–brain barrier; CD8^+^, cluster of differentiation 8-positive (cytotoxic T cells); DAMP, damage-associated molecular pattern; DSF, disulfiram; EMT, epithelial–mesenchymal transition; FIN56, ferroptosis inducer 56; GBM, glioblastoma; GPX4, glutathione peroxidase 4; GSH, glutathione; IDH1, isocitrate dehydrogenase 1; IFN-γ, interferon gamma; NRF2, nuclear factor erythroid 2-related factor 2; PD-L1, programmed death-ligand 1; ROS, reactive oxygen species; RSL3, RAS-selective lethal 3; TMZ, temozolomide; xCT/SLC7A11, cystine/glutamate antiporter. To date, the summarized combinations demonstrate enhanced ferroptosis sensitization and reversal of treatment resistance; dose-sparing and toxicity-reduction benefits have not yet been established and require dedicated studies. References in brackets correspond to citations in the main text.

## Data Availability

No new data were created or analyzed in this study. Data sharing is not applicable to this article.

## Abbreviations

The following abbreviations are used in this manuscript:

ABCC1	ATP-binding cassette subfamily C member 1
ACOD1	aconitate decarboxylase 1
ACSL4	acyl-CoA synthetase long-chain family member 4
AGPAT3	1-acylglycerol-3-phosphate O-acyltransferase 3
AKT	protein kinase B
ALOX	arachidonate lipoxygenases
ALOXE3	arachidonate lipoxygenase 3
ARE	antioxidant response element
ARG1	arginase 1
ATF	activating transcription factor
ATP	adenosine triphosphate
BER	base excision repair
BBB	blood–brain barrier
BRAF	B-Raf proto-oncogene serine/threonine kinase
CAR-NK	chimeric antigen receptor natural killer
CAR-T	chimeric antigen receptor T
CBTRUS	Central Brain Tumor Registry of the United States
CD4	cluster of differentiation 4
CD8	cluster of differentiation 8
CD274	programmed death-ligand 1
cDC1	conventional dendritic cells type 1
CNS	central nervous system
CoA	coenzyme A
CoQ10	coenzyme Q10
CoQ10H2	reduced coenzyme Q10 (ubiquinol)
DAMPs	damage-associated molecular patterns
DCs	dendritic cells
DHA	dihydroartemisinin
DHODH	dihydroorotate dehydrogenase
DSF	disulfiram
EGFR	epidermal growth factor receptor
ERK	extracellular signal-regulated kinase
FasL	Fas ligand
FSP1	ferroptosis suppressor protein 1
FTH1	ferritin heavy chain 1
FTL	ferritin light chain
GBM	glioblastoma
GM-CSF	granulocyte-macrophage colony-stimulating factor
GPX4	glutathione peroxidase 4
GSH	glutathione
HAMP	hepcidin antimicrobial peptide
HFE	homeostatic iron regulator
HIF-1α	hypoxia-inducible factor 1 alpha
HMGB1	high mobility group box 1
HMOX1	heme oxygenase 1
HRE	hypoxia response element
HSPA5	heat shock protein family A member 5
IC50	half-maximal inhibitory concentration
ICD	immunogenic cell death
IDH1	isocitrate dehydrogenase 1
IFN-γ	interferon gamma
IL	interleukin
iNOS	inducible nitric oxide synthase
KEAP1	Kelch-like ECH-associated protein 1
LGG	low-grade glioma
LMP	lysosomal membrane permeabilization
lncRNA	long non-coding RNA
LOX	lipoxygenase
LPCAT	lysophosphatidylcholine acyltransferase
m6A	N6-methyladenosine
MAZ	MYC-associated zinc finger protein
MDA	malondialdehyde
MDSCs	myeloid-derived suppressor cells
METTL3	methyltransferase-like 3
MGMT	O6-methylguanine-DNA methyltransferase
miR-491-5p	microRNA-491-5p
MLH1	MutL homolog 1
MMR	mismatch repair
mRNA	messenger RNA
MSH6	MutS homolog 6
mTOR	mechanistic target of rapamycin
NCOA4	nuclear receptor coactivator 4
NF-κB	nuclear factor kappa B
NK	natural killer
NKG2D	natural killer group 2D receptor
NOX4	NADPH oxidase 4
NRF2	nuclear factor erythroid 2-related factor 2
NUAK2	NUAK family kinase 2
OTUB1	OTU deubiquitinase ubiquitin aldehyde binding 1
PBMCs	peripheral blood mononuclear cells
PD-1	programmed cell death protein 1
PD-L1	programmed death-ligand 1
PEBP1	phosphatidylethanolamine-binding protein 1
PERK	protein kinase R-like endoplasmic reticulum kinase
PGE2	prostaglandin E2
PI3K	phosphoinositide 3-kinase
PL•	phospholipid radical
PLOO•	phospholipid peroxyl radical
PLOOH	phospholipid hydroperoxide
PMS2	PMS1 homolog 2
PUFAs	polyunsaturated fatty acids
PUFA-CoA	polyunsaturated fatty acyl-CoA
PUFA-PL	polyunsaturated fatty acid-containing phospholipids
RND1	Rho family GTPase 1
ROS	reactive oxygen species
RSL3	Ras-selective lethal 3
RTAs	radical-trapping antioxidants
SAT1	spermidine/spermine N1-acetyltransferase 1
SFAs	saturated fatty acids
SHP-1	Src homology region 2 domain-containing phosphatase-1
siRNA	small interfering RNA
SIRT3	sirtuin 3
SLC3A2	solute carrier family 3 member 2
SLC7A11	solute carrier family 7 member 11
SOD1	superoxide dismutase 1
STAT3	signal transducer and activator of transcription 3
System Xc^−^	cystine/glutamate antiporter system
TAMs	tumor-associated macrophages
TERT	telomerase reverse transcriptase
TF	transferrin
TFEB	transcription factor EB
TFR1	transferrin receptor 1
TFRC	transferrin receptor
TFR2	transferrin receptor 2
TGF-β	transforming growth factor beta
TGM2	transglutaminase 2
TME	tumor microenvironment
TMZ	temozolomide
TNF-α	tumor necrosis factor alpha
TP53	tumor protein p53
Tregs	regulatory T cells
TRIM7	tripartite motif containing 7
TUG1	taurine upregulated gene 1
VDAC2	voltage-dependent anion channel 2
VEGF	vascular endothelial growth factor
VKH2	reduced vitamin K hydroquinone
xCT	cystine/glutamate antiporter light chain
